# Novel Tet(L) Efflux Pump Variants Conferring Resistance to Tigecycline and Eravacycline in Staphylococcus Spp.

**DOI:** 10.1128/Spectrum.01310-21

**Published:** 2021-12-08

**Authors:** Nannan Wang, Dexi Li, Stefan Schwarz, Shangshang Qin, Hong Yao, Xiang-Dang Du

**Affiliations:** a College of Veterinary Medicine, Henan Agricultural University, Zhengzhou, People’s Republic of China; b Institute of Microbiology and Epizootics, Centre for Infection Medicine, Department of Veterinary Medicine, Freie Universität Berlin, Berlin, Germany; c School of Pharmaceutical Sciences, Zhengzhou University, Zhengzhou, People’s Republic of China; National University Hospital

**Keywords:** Tet(L), efflux pump, variant, tigecycline, *Staphylococcus* spp., *Staphylococcus aureus*

## Abstract

Tigecycline is regarded as one of the few important last-resort antibiotics to treat complicated skin and intra-abdominal infections. Members of the genus Staphylococcus are zoonotic pathogens and pose a serious threat to public health. Tigecycline resistance in this species appears to be a rare phenomenon, and the mechanisms underlying tigecycline resistance have not been fully elucidated. Here, we report two novel variants of the *tet*(L) gene in Staphylococcus spp. from swine in China, designed as *tet*(L)_F58L_ and *tet*(L)_A117V_. The *tet*(L)_F58L_ was located within a 18,720 bp chromosomal multidrug resistance gene cluster flanked by two copies of IS*257* in Staphylococcus cohnii 11-B-312, while the *tet*(L)_A117V_ was located on a 6,292 bp plasmid in *S. haemolyticus* 11-B-93, which could be transferred to S. aureus by electrotransformation. Cloning of each of the two *tet*(L) variants into S. aureus RN4220 showed 16- or 8-fold increases in the minimal inhibition concentrations (MICs), which can fully confer the resistance to tigecycline (MICs from 0.125 to 2 mg/liter) and eravacycline (MICs from 0.125 to 1 or 2 mg/liter), but no increase in the MICs of omadacycline, compared with the MICs of the recipient strain S. aureus RN4220. In the *in vivo* murine sepsis and in the murine pneumonia models, an increase in CFU of S. aureus 29213_pT93 carrying the *tet*(L)_A117V_ was seen despite tigecycline treatment. This observation suggests that the *tet*(L)_A117V_ and its associated gene product compromise the efficacy of tigecycline treatment *in vivo* and may lead to clinical treatment failure. Our finding, that novel Tet(L) efflux pump variants which confer tigecycline and eravacycline resistance have been identified in Staphylococcus spp., requires urgent attention.

**IMPORTANCE** Tigecycline and eravacycline are both important last-resort broad spectrum antimicrobial agents. The presence of novel Tet(L) efflux pump variants conferring the resistance to tigecycline and eravacycline in Staphylococcus spp. and its potential transmission to S. aureus will compromise the efficacy of tigecycline and eravacycline treatment for S. aureus associated infection *in vivo* and may lead to clinical treatment failure.

## INTRODUCTION

The emergence and wide dissemination of multidrug-resistant bacteria pose serious threats to public health worldwide by compromising the efficacy of antimicrobial treatments in human and veterinary medicine (1). Tigecycline is recognized as one of the few last-resort antibiotics. It is a 9-t-butylglycylamido derivative of minocycline, belonging to glycylcycline class of antimicrobial agents (2). Tigecycline is a protein synthesis inhibitor and has been approved by the FDA for the treatment of complicated skin and skin-structure infections as well as complicated intra-abdominal infections in 2005 (3). Tigecycline has been only authorized for use in human medicine worldwide (4).

During recent years, tigecycline resistance has emerged and mostly been identified in Gram-negative bacteria, especially in Enterobacterales and Acinetobacter isolates (5–8). A number of studies have revealed that decreased susceptibility to tigecycline is primarily due to overexpression of efflux pumps of the resistance-nodulation-cell division (RND) superfamily (e.g., AcrAB-TolC and AdeABC) (9–11), plasmid-borne efflux pump TmexCD1-ToprJ (12), mutations in the genes for the ribosomal proteins, such as S10 (13), and/or enzymatic inactivation (e.g., via *tet*[X] variants) (5, 6). In our previous studies, specific efflux system encoding genes for tigecycline resistance were also identified, such as a *tet*(A) variant in Klebsiella pneumoniae and a *tet*(L) variant in Escherichia coli (14, 15).

Compared with Gram-negative bacteria, tigecycline resistance is rarely reported in Gram-positive bacteria and the mechanisms underlying tigecycline resistance have not been fully elucidated, especially in Staphylococcus spp., which can cause a variety of diseases in both animals and humans (16, 17). In Staphylococcus spp., only the overexpression of the multidrug and toxin extrusion family efflux pump MepA and mutations in the *rpsJ* gene coding for the ribosomal protein S10 have been shown so far to be associated with decreased susceptibility to tigecycline (13, 18–20). The gene *tet*(L), which can export tetracycline and doxycycline, but not minocycline and tigecycline, was first described in the genus Staphylococcus in 1992 (21). During the following years, this gene was detected in a variety of staphylococci from different animal species, including pigs (22, 23). In staphylococci, the *tet*(L) gene was commonly located on plasmids that differed in size, structure, and co-located antimicrobial resistance genes (21, 24–26).

In this study, two novel *tet*(L) gene variants conferring resistance to tigecycline and eravacycline were identified in Staphylococcus spp. of swine origin. In addition, the effect on the efficacy of tigecycline treatment in the presence of one of these *tet*(L) variants in murine sepsis and pneumonia models was evaluated *in vivo*.

## RESULTS AND DISCUSSION

### Two novel *tet*(L) variants were identified in porcine staphylococci.

A total of 362 nonduplicate porcine Staphylococcus spp. isolates were subjected to antimicrobial susceptibility testing (AST) during routine antibiotic resistance surveillance. According to the interpretation criteria of minimal inhibition concentrations (MICs) in the version 11.0 issued by EUCAST in 2021, the breakpoint of tigecycline for Staphylococcus spp. is 0.5 mg/liter, which means that a MIC greater than 0.5 mg/liter is considered resistant. Two isolates (Staphylococcus cohnii 11-B-312 and *S. haemolyticus* 11-B-93) displayed resistance to tigecycline (4 mg/liter) in addition to tetracycline, erythromycin, chloramphenicol, and ampicillin resistance (Table 1). Screening for potential tigecycline resistance determinants [mutations in *rpsJ*, *mepA*, mutated *tet*(A), *tet*(L) and *tet*(X) genes] by PCR and sequencing revealed that only *tet*(L) mutations, but no other acquired *tet* genes or mutations were identified in these two tigecycline-resistant Staphylococcus isolates. Further analysis revealed that compared with the reference staphylococcal *tet*(L) gene from plasmid pG38 (GenBank accession number RCDF01000030.1), the amino acid substitution F58L was present in the deduced Tet(L) sequence of S. cohnii 11-B-312, while the substitution A117V was detected in S. haemolyticus 11-B-93. A BLASTp search for these two Tet(L) variants in GenBank database retrieved no same mutations in Tet(L). Consequently, the two Tet(L) proteins were designed Tet(L)_F58L_ and Tet(L)_A117V_, respectively.

**TABLE 1:** Minimal inhibition concentrations (MICs) of antibiotics for various strains

Staphylococcal strains	Description	MICs (mg/L)							
		TIG^*a*^	ERA	OMA	TET	DOX	ERY	CHL	AMP
11-B-312	Staphylococcus cohnii isolate	4	4	1	32	8	>512	128	32
11-B-93	Staphylococcus haemolyticus isolate	4	4	0.5	64	32	>512	64	16
T93	S. aureus RN4220 transformant harboring pT93	2	2	0.25	64	16	>512	8	4
RN4220	Recipient strain	0.125	0.125	0.125	<1	<1	<1	4	2
RN4220+pLI50	RN4220 transformed with plasmid pLI50^*b*^	0.125	0.125	0.125	<1	<1	<1	128	2
RN4220+pLI50_reference *tet*(L)	RN4220 transformed with the recombinant plasmid pLI50 that carries the reference *tet*(L) gene	0.125	0.125	0.125	32	4	<1	128	2
RN4220+pLI50_*tet*(L)_F58L_	RN4220 transformed with the recombinant plasmid pLI50 that carries the *tet*(L)_F58L_	2	2	0.125	32	8	<1	128	2
RN4220+pLI50_*tet*(L)_A117V_	RN4220 transformed with the recombinant plasmid pLI50 that carries the *tet*(L)_A117V_	2	1	0.125	64	8	<1	128	2
DH5α	Recipient strain	0.25	0.06	0.5	0.5	0.5	^*c*^	4	
DH5α+ pLI50	DH5α transformed with plasmid pLI50	0.25	0.06	0.5	1	1		32	
DH5α+ pLI50_reference *tet*(L)	DH5α transformed with the recombinant plasmid pLI50 that carries the reference *tet*(L) gene	0.5	0.125	0.5	32	32		32	
DH5α+ pLI50_*tet*(L)_F58L_	DH5α transformed with the recombinant plasmid pLI50 that carries the *tet*(L)_F58L_	0.5	0.125	0.5	32	16		32	
DH5α+ pLI50_*tet*(L)_A117V_	DH5α transformed with the recombinant plasmid pLI50 that carries the *tet*(L)_A117V_	0.5	0.125	0.5	32	16		32	
29213_pT93	S. aureus ATCC 29213 transformant harboring pT93	1	2	0.125	64	8	>512	16	
ATCC 29213	Recipient strain/quality control strain for AST	<0.125	0.125	0.125	<1	<1	<1	8	

a TIG, tigecycline; ERA, eravacycline; OMA, omadacycline; TET, tetracycline; DOX, doxycycline; ERY, erythromycin; CHL, chloramphenicol; AMP, ampicillin.

b Plasmid pLI50 is empty vector.

c Not determined.

### The location and genetic context of two novel *tet*(L) variants.

**FIG 1:**
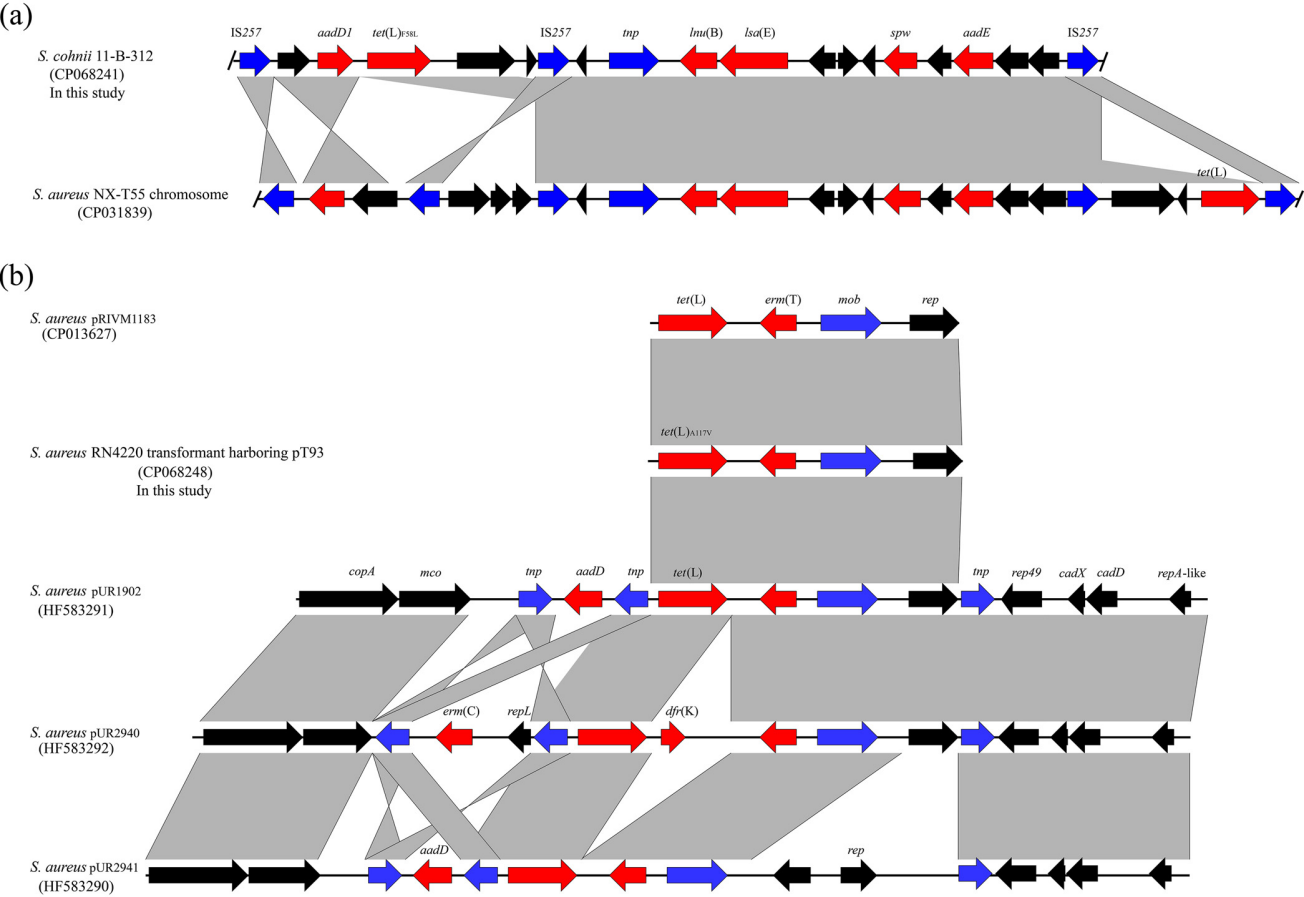
Genetic structure of the *tet*(L) variants in this study and its comparison with the similar region in those deposited in GenBank. (a) Genetic structure of the chromosomal *tet*(L)_F58L_ in S. cohnii 11-B-312 and its comparison with the similar region in S. aureus NX-T55. (b) Genetic structure of plasmid-borne *tet*(L)_A117V_ in transformant and its comparison with the similar regions in other plasmids. The positions and transcriptional directions of the predicted ORFs are indicated by arrows. The antimicrobial resistance genes are colored in red, and insertion sequences in blue. Genes with predicted functions are showed in black. Regions of >99% homology are denoted by gray shading.

Whole gene sequencing for *tet*(L)_F58L_- and *tet*(L)_A117V_-carrying strains were performed. Sequence analysis revealed that the *tet*(L)_F58L_, along with the pleuromutilin-lincosamide-streptogramin A resistance gene *lsa*(E), the lincosamide resistance gene *lnu*(B), and the aminoglycoside resistance genes *aadD*, *spw*, and *aadE*, was located within a 18,720 bp chromosomal multidrug-resistance gene cluster flanked by two copies of IS*257* in the same orientation in *S. cohnii* 11-B-312 (Fig. 1a). The BLAST analysis revealed that this multidrug-resistance gene cluster showed 100% query coverage and 99.97% identity with that in the *tet*(L)-carrying chromosomal DNA of S. aureus NX-T55 (GenBank accession number CP031839) (Fig. 1a).

The *tet*(L)_A117V_, along with the macrolide-lincosamide-streptogramin B (MLS_B_) resistance gene *erm*(T), was located on a small plasmid of 6,292 bp in S. haemolyticus 11-B-93 (Fig. 1b). This plasmid was designated pT93 and proved to be transferable by electrotransformation. Using the sequence of plasmid pT93 for a GenBank database search, several *tet*(L)-carrying plasmids with similar sequences matched the query sequence, namely, pRIVM1183 (CP013627; nucleotide sequence identity, 98.58%; query coverage, 98%), pUR1902 (HF583291; nucleotide sequence identity, 98.54%; query coverage, 98%), pUR2940 (HF583292; nucleotide sequence identity, 92.12%; query coverage, 98%) and pUR2941 (HF583290; nucleotide sequence identity, 98.58%; query coverage, 80%). Of them, the *tet*(L)-carrying plasmid pRIVM1183 with a size of 6,183 bp was from a methicillin-resistant S. aureus of human origin (Fig. 1b) (27). The sequence similarity among these plasmids suggested that they can be transferred between coagulase-negative staphylococci and S. aureus under natural conditions. Under laboratory conditions, we showed that plasmid pT93 carrying the *tet*(L)_A117V_ could be transferred into S. aureus, where it expressed tigecycline and MLS_B_ resistance.

### The role of *tet*(L) variants in conferring resistance to tigecycline.

To confirm the role of *tet*(L) variants in conferring tigecycline resistance in Staphylococcus spp., the intact copies of *tet*(L)_F58L_ and *tet*(L)_A117V_ including their respective putative promoters were cloned into the pLI50 vector, and then introduced into S. aureus RN4220. The constructs, both RN4220+pLI50_*tet*(L)_F58L_ and RN4220+pLI50_*tet*(L)_A117V_ showed 16-fold increases in the MICs of tigecycline from 0.125 mg/liter to 2 mg/liter, compared with those of S. aureus RN4220 and S. aureus RN4220+pLI50_reference *tet*(L) (Table 1). Therefore, both *tet*(L) variants *tet*(L)_F58L_ and *tet*(L)_A117V_ can fully confer resistance to tigecycline as the MICs breakpoint of tigecycline for Staphylococcus spp. is 0.5 mg/liter according to the interpretation criteria of MICs in the version 11.0 issued by EUCAST in 2021. In addition, RN4220+pLI50_*tet*(L)_F58L_ and RN4220+pLI50_*tet*(L)_A117V_ displayed 16-fold and 8-fold increases in the MICs of eravacycline, reaching 2 mg/liter and 1 mg/liter, respectively (Table 1). Eravacycline is a newly FDA-approved drug of the glycylcycline subclass with broad-spectrum antimicrobial activity against most Gram-positive and Gram-negative bacteria. As the MICs breakpoint of eravacycline for S. aureus is 0.25 mg/liter, both *tet*(L) variants *tet*(L)_F58L_ and *tet*(L)_A117V_ can also fully confer resistance to eravacycline. However, RN4220+pLI50_*tet*(L)_F58L_ and RN4220+pLI50_*tet*(L)_A117V_ displayed no increases in the MICs of omadacycline, a unique first-in class aminomethylcycline (Table 1). In addition, the function of two *tet*(L) variants was also evaluated in Gram-negative bacteria E. coli. DH5α+pLI50_*tet*(L)_F58L_ and DH5α+pLI50_*tet*(L)_A117V_ displayed no increases in the MICs of tigecycline, eravacycline and omadacycline, compared with DH5α+pLI50_reference *tet*(L) (Table 1). These results strongly revealed that the *tet*(L) variants *tet*(L)_F58L_ and *tet*(L)_A117V_ were responsible for the significantly elevated MICs to tigecycline and eravacycline in Staphylococcus spp.

Tet(L), composed of 14 transmembrane segments, belongs to the major facilitator superfamily efflux pump that exports tetracycline but neither minocycline nor glycylcyclines from the bacterial cell. Tet(L) was first found in *Bacillus* in 1988 (28) and staphylococcal *tet*(L)-carrying plasmid pSTE1 from a porcine Staphylococcus hyicus isolate was identified in 1992 (21). The *tet*(L) that reported in plasmid pG38 in S. aureus (GenBank accession number RCDF 01000030.1) could confer resistance to tetracycline but not to tigecycline (26). It was regarded as a reference to confirm the function of *tet*(L)_F58L_ and *tet*(L)_A117V_ for tigecycline resistance in Staphylococcus spp. in this study, though the comparison of the Tet(L) (RCDF 01000030.1) and the earlier reported Tet(L) (M11036) in *Bacillus* revealed an L363S residue substitution.

Recently, a Tet(L) variant was identified, in which multiple amino acid substitutions/deletions, such as the loss of the first codon, N2M, T3K, S4C, and Y5N were detected. This variant exhibited no elevated tigecycline MICs in Campylobacter but showed a 4-fold increased tigecycline MICs in E. coli (15). The two novel Tet(L) variants with substitutions F58L and A117V, respectively, identified in this study conferred significantly elevated tigecycline and eravacycline MICs (8- or 16-fold) in S. aureus but displayed no increase in the MICs of tigecycline, eravacycline, and omadacycline in E. coli (Table 1).

The information for crystal structure of Tet(L) is currently not available in the Protein Data Bank database. Therefore, we established the predicted structure of Tet(L) by homology modeling, based on the crystal structure of YajR (PDB ID code 3WDO), which exhibits the most greatest identity with Tet(L) (29). The result showed that F58L was localized in helices and A117V in loop, respectively, which may associate with its role in substrate binding or transport in Tet(L) (data not shown). However, because of the unavailability of crystal structure of Tet(L), the explanations about how the F58L and A117V mutation precisely influence the structure and function of Tet(L) need further studies.

In addition, the copy number of the resistance genes affects gene expression, which leads to the elevated MICs (30, 31). In this study, a medium-copy number plasmid pLI50 (15–20 per cell) was used. We cloned the reference *tet*(L), the variants *tet*(L)_F58L_ and *tet*(L)_A117V_ into this same vector pLI50, respectively, and then transform them into the same recipient to eliminate the influence of the copy number of these resistance genes on gene expression to some extent. As both tigecycline and eravacycline are important options for the treatment of S. aureus infections, the emergence of the *tet*(L) variants in Staphylococcus spp. might impair the efficacy of treatment with these two antibiotics.

### Impact of *tet*(L)_A117V_ on tigecycline treatment in the murine sepsis and pneumonia models.

Murine sepsis and pneumonia infection models were used to evaluate the effect of *tet*(L)_A117V_-mediated tigecycline resistance on the efficacy of tigecycline treatment *in vivo*. The *tet*(L)_A117V_-carrying plasmid pT93 was introduced into S. aureus ATCC 29213, designed 29213_pT93, and the S. aureus ATCC 29213 was used as control (Table 1).

**FIG 2:**
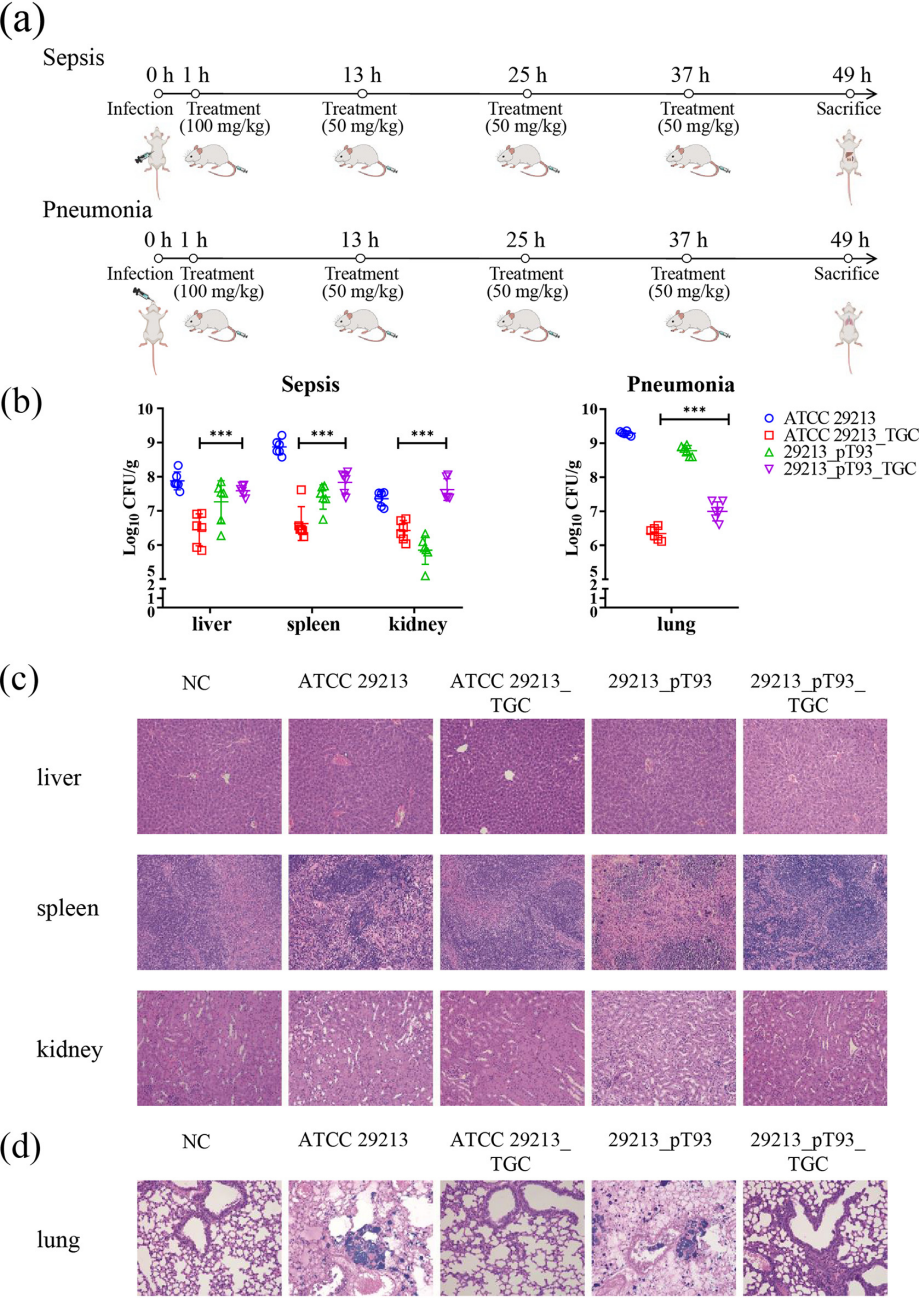
Murine sepsis and pneumonia infection models. (a) The schedule for infection, treatment and sacrifice in murine sepsis- and pneumonia- infection models. (b) CFU counts of S. aureus ATCC 29213 and S. aureus 29213_pT93 in the liver, spleen and kidney, respectively, in murine sepsis infection model. CFU counts of S. aureus ATCC 29213 and S. aureus 29213_ pT93 in lung in murine pneumonia model. (c) H&E staining results of tissues (liver, spleen and kidney) from the murine sepsis model after infected with S. aureus ATCC 29213 and S. aureus 29213_ pT93 and their respective tigecycline treatment groups. (d) H&E staining results of tissues (lung) from the murine pneumonia model after infected with S. aureus ATCC 29213 and S. aureus 29213_ pT93 and their respective tigecycline treatment groups. Data are mean ± standard deviation. *P* values were calculated using an independent two sample T-test for the log-transformed difference in CFU after treatment (***, *P* < 0.001).

In the murine sepsis model, body weight loss and diarrhea occurred after the intraperitoneal injection of S. aureus ATCC 29213 or S. aureus 29213_pT93 at an initial dose of 10^8^ CFU. Tigecycline significantly reduced the S. aureus ATCC 29213 load in the liver, spleen and kidney by 0.93–2.24 log orders of magnitude (Fig. 2b). In contrast, the CFU counts of 29213_pT93 in the liver, spleen and kidney were increased, especially in the kidney (1.77 log orders of magnitude), over a 48-h period after tigecycline treatment (Fig. 2b). Hematoxylin and eosin (H&E) staining results of the mice in the sepsis groups (infection by ATCC 29213 and 29213_pT93) showed that parenchymatous organs appeared degenerated and infiltrated with inflammatory cells, compared with the control group (Fig. 2c). Tigecycline was more effective in ATCC 29213 infection group than in 29213_pT93 infection group (Fig. 2c).

In the murine pneumonia model, S. aureus ATCC 29213 was reduced by 2.95 log orders of magnitude over a 48-h period after tigecycline treatment, compared with 1.78-log decrease in 29213_pT93 (Fig. 2b). H&E staining results for murine lungs in the pneumonia model (infection by S. aureus ATCC 29213 and S. aureus 29213_pT93) indicated that the alveolar structure had disappeared, and a large number of bacterial clumps were scattered, accompanied by inflammatory cell infiltration, which remained even after tigecycline treatment in 29213_pT93 infection group (Fig. 2d).

The *in vivo* experiments stated above suggested that the presence of the *tet*(L)_A117V_ compromises the efficacy of tigecycline treatment *in vivo* and may lead to tigecycline treatment failure in humans.

## CONCLUSIONS

In conclusion, we report the presence of two novel *tet*(L) variants in Staphylococcus spp. from swine, which are able to confer full resistance to tigecycline and eravacycline whether in native host or in the S. aureus host. The presence of these *tet*(L) variants may compromise the efficacy of tigecycline treatment as shown in the murine sepsis and pneumonia model, which eventually may lead to clinical treatment failure. From the perspective of “One Health,” measures to monitor and control the dissemination of the novel *tet*(L) variants in both animal and human clinical strains are needed.

## MATERIALS AND METHODS

### Bacterial strains and AST.

A total of 362 nonduplicate Staphylococcus spp. isolates were investigated in this study. These isolates were collected in 2019 from nasal swabs of swine in Henan province/China and were cultured on Staphylococcus chromogenic medium. After incubation at 37°C for 24–36 h, colonies were selected for 16S rRNA sequencing as described previously (32). Staphylococcus aureus RN4220 served as recipient strain in electrotransformation experiments.

AST was performed by broth microdilution according to the recommendations given in document M100 (30^th^ edition) issued by the Clinical and Laboratory Standards Institute (CLSI) (33). S. aureus ATCC 29213 served as the quality control strain. AST results were interpreted according to CLSI, except for tigecycline and eravacycline, where the interpretation criteria of MICs issued by the European Committee on AST (EUCAST) (http://www.eucast.org/clinical_breakpoints/) was used.

### PCR analysis.

Staphylococcus cohnii 11-B-312 and Staphylococcus haemolyticus 11-B-93 with tigecycline MICs of 4 mg/liter were screened for the presence of the *tet*(A), *tet*(K), *tet*(Y), *tet*(M), *tet*(L) and *tet*(X) genes by PCR using the primers listed in Table S1. The PCR mixture was composed of 12.5 μl of *Ex Taq* (TaKaRa, Dalian, China), 0.5 μl of each primer, 0.5 μl of chromosomal DNA template, and 11 μl of sterile distilled water. All PCR products were subjected to Sanger sequencing.

### Transfer experiments.

Whole cell DNA of S. cohnii 11-B-312 and plasmid DNA of S. haemolyticus 11-B-93 were extracted using the Gentra Puregene Yeast/Bact. kit or the Qiagen plasmid extraction midi kit (both Qiagen, Hilden, Germany). The plasmid DNA of strain 11-B-93 was introduced into the recipient strain S. aureus RN4220 and the QC strain S. aureus 29213 by electrotransformation as previously described (26). Brain heart infusion agar supplemented with 0.8 mg/liter tigecycline was used for screening transformants. Colonies that grew on selective plates after incubation for 16–24 h at 37°C were further confirmed by AST and PCR analysis.

### Whole genome sequencing.

Whole genome DNA of S. cohnii 11-B-312 and the transfomant of S. haemolyticus 11-B-93 (transformant S. aureus T93) were sequenced by the PacBio RS and Illumina MiSeq platforms. The PacBio sequence reads were assembled with HGAP4 and CANU (version 1.6) and corrected by Illumina MiSeq with pilon (version 1.22). The prediction of ORFs and their annotations were performed using Glimmer 3.0. Insertion sequences were predicted by using ISfinder (www-is.biotoul.fr). Resistance genes were identified with ResFinder (version 4.0) (34).

### Functional cloning of the *tet*(L) variants.

To confirm whether the *tet*(L) variants confer tigecycline resistance, *tet*(L)_F58L_ and *tet*(L)_A117V_ were separately cloned into the E. coli-S. aureus shuttle vector pLI50 (35). Briefly, the plasmid pLI50 was linearized by BamHI-digesting. A pair of primers (Table S1 in the supplemental material) was designed to amplify the complete copies of both *tet*(L) variants and their putative promoter from *S. cohnii* 11-B-312 and the transformant S. aureus T93, respectively, using the online assembly tool NEBuilder (New England Biolabs, Ipswich, MA). The same cloning procedure was conducted for the reference *tet*(L) gene from the S. aureus plasmid pG38 (26). Then, the amplicons were ligated into the linearized plasmid pLI50 by using the NEBuilder HiFi DNA Assembly Master Mix (New England Biolabs). Each of the recombinant plasmids, pLI50-*tet*(L)_F58L_ and -*tet*(L)_A117V_, but also pLI50-reference *tet*(L), were subsequently electrotransformed into S. aureus RN4220, respectively, as described previously (26).

### Murine sepsis and pneumonia model.

All animal experiments were conducted in accordance with the approved guidelines of the Institutional Animal Care and Use Committee. Seven- to 9-week-old BALB/c female mice weighing 15–18 g served for the sepsis model and SPF level KM female mice for the pneumonia model according to the methods described previously (36, 37). They were purchased from Henan Hua Xing Laboratory Animal Co., Ltd. (Zhengzhou, China; HXDW20010004). The mice were randomly divided into five groups, including Group ATCC 29213 (infection with S. aureus ATCC 29213), Group 29213_TGC (infection with S. aureus ATCC 29213 and treated with tigecycline), Group 29213_pT93 (infection with S. aureus 29213_pT93), Group 29213_pT93_TGC (infection with 29213_pT93 and treated with tigecycline), and Group NC, which represented the negative control (no infection, no treatment). Each group contained six mice.

Prior to tigecycline injection, BALB/c female mice were intraperitoneally injected with bacterial suspension (1 × 10^8^ CFU of the S. aureus 29213_pT93) and KM female mice were infected by bacteria though the nasal cavity (5 × 10^7^ CFU of S. aureus 29213_pT93). The mice were treated with tigecycline by subcutaneous injection at an initial dose of 100 mg kg^−1^ 1 h after the infection and then subsequently with 50 mg kg^−1^ tigecycline every 12 h, which is consistent with the treatment levels of tigecycline used in humans. The mice were euthanized and were dissected after 48 h (Fig. 2a). The liver, spleen and kidney tissues were collected for the sepsis model and the lung tissues for the pneumonia model. Parts of all organs were aseptically transferred into 1 ml sterile PBS on ice and homogenized for calculating the CFU of bacteria in each organ. At the same time, H&E staining was also performed for observing liver, spleen and kidney lung tissue lesions.

### Data availability.

The genomic sequence of *S. cohnii* 11-B-312 and the plasmid sequence of S. aureus T93 plasmid, pT93, determined in this study have been deposited in GenBank under accession numbers CP068241 and CP068248, respectively.
