# Supplementary material for: Novel Tet(L) Efflux Pump Variants Conferring Resistance to Tigecycline and Eravacycline in Staphylococcus Spp
Source: Microbiol Spectr. 2021 Dec 8;9(3):e01310-21. doi: 10.1128/Spectrum.01310-21 (PMC8653819; doi:10.1128/Spectrum.01310-21)
Supplement: SUPPLEMENTAL FILE 1 — Supplemental material. Download SPECTRUM01310-21_Supp_1_seq5.pdf, PDF file, 0.1 MB [file spectrum01310-21_supp_1_seq5.pdf]

## Supplementary material

Table S1. Primers used for PCR in this study.

| Resistance gene | Primer sequence (5'-3') | Reference |
|---|---|---|
| *tet*(A) | GCTACATCCTGCTTGCCTTC<br>CATAGATCGCCGTGAAGAGG | [1] |
| *tet*(K) | TCGATAGGAACAGCAGTA<br>CAGCAGATCCTACTCCTT | [1] |
| *tet*(Y) | ATTTGTACCGGCAGAGCAAAC<br>GGCGCTGCCGCCATTATGC | [2] |
| *tet*(M) | AGTTTTAGCTCATGTTGATG<br>TCCGACTATTTAGACGACGG | [1] |
| *tet*(L) | GTTGCGCGCTATATTCCAAA<br>TTAAGCAAACTCATTCCAGC | this study |
| *tet*(X3) | TAATGGCGGGACATCAGG<br>AGGCGACATCAAATGAGCAG | [3] |
| *tet*(X4) | CCGATATTCATCATCCAGAGG<br>CGCTTACTTTTCCAAGACTTACCT | [3] |
| *tet*(L) variants | GAATTCGAGCTCGGTACCCGGGTTTCAACAAACGGGCCATATTG<br>CCTGCAGGTCGACTCTAGAGGATCCCCGAAAGCCAGACTCAGC | this study |

## Reference

[1] Ng, L.K., Martin, I., Alfa, M., Mulvey, M., 2001. Multiplex PCR for the detection of tetracycline resistant genes. Mol. Cell. Probes 15, 209–215.

[2] S N AB, B M AM, A S AA, A E E, I Y M. *Escherichia coli* tetracycline efflux determinants in relation to tetracycline residues in chicken. Asian Pac J Trop Med. 2013 Sep;6(9):718-22

[3] He T, Wang R, Liu D, Walsh TR, Zhang R, Lv Y, Ke Y, Ji Q, Wei R, Liu Z, Shen Y, Wang G, Sun L, Lei L, Lv Z, Li Y, Pang M, Wang L, Sun Q, Fu Y, Song H, Hao Y, Shen Z, Wang S, Chen G, Wu C, Shen J, Wang Y. 2019. Emergence of plasmid-mediated high-level tigecycline resistance genes in animals and humans. Nat Microbiol 4:1450-1456.
